# A survey on table tolerances and couch overrides in radiotherapy

**DOI:** 10.1120/jacmp.v17i6.6261

**Published:** 2016-11-08

**Authors:** Bonnie Chinsky, Rakesh Patel, Joshua Panfil, Murat Surucu, John C. Roeske

**Affiliations:** ^1^ Department of Radiation Oncology Loyola University Medical Center Maywood IL USA

**Keywords:** quality assurance, patient safety, radiotherapy, treatment overrides, couch overrides

## Abstract

The purpose of this study was to survey current departmental policies on treatment couch overrides and the values of table tolerances used clinically. A 25‐question electronic survey on couch overrides and tolerances was sent to full members of the American Association of Physicists in Medicine (AAPM). The first part of the survey asked participants if table overrides were allowed at their institution, who was allowed to perform these overrides, and if imaging was required with overrides. The second part of the survey asked individuals to provide table tolerance data for the following treatment sites: brain/head and neck (H&N), lung, breast, abdomen/pelvis and prostate. Each site was further divided into IMRT/VMAT and 3D conformal techniques. Spaces for free‐text were provided, allowing respondents to enter any table tolerance data they were unable to specify under the treatment sites listed. A total of 361 individuals responded, of which approximately half participated in the couch tolerances portion of the survey. Overall, 86% of respondents’ institutions allow couch tolerance overrides at treatment. Therapists were the most common staff members permitted to perform overrides, followed by physicists, dosimetrists, and physicians, respectively. Of the institutions allowing overrides, 34% reported overriding daily. More than half of the centers document the override and/or require a setup image to radiographically verify the treatment site. With respect to table tolerances, SRS/SBRT table tolerances were the tightest, while clinical setup table tolerances were the largest. There were minimal statistically significant differences between IMRT/VMAT and 3D conformal table tolerances. Our results demonstrated that table overrides are relatively common in radiotherapy despite being a potential safety concern. Institutions should review their override policy and table tolerance values in light of the practices of other institutions. Careful attention to these matters is crucial in ensuring the safe and accurate delivery of radiotherapy.

PACS number(s): 87.55.N‐, 87.55.Qr, 87.55.T‐

## I. INTRODUCTION

In recent years, publications in the popular press have pointed out serious errors in the delivery of radiotherapy that have resulted in adverse patient outcomes.^(1)^ As a result of these findings, a number of safety initiatives have been implemented both locally and nationally. For example, many institutions have adopted daily treatment “time outs,” checklists, and quality assurance rounds.^2^, ^3^, ^4^, ^5^, ^6^ Larger hospital systems have provided an infrastructure for sharing and disseminating critical information related to patient safety, “near misses,” and treatment errors.^3^, ^4^ Guidelines have also been provided nationally and internationally by ASTRO, AAPM, and other organizations, through “Safety is no Accident,” as well as the IAEA.^7^, ^8^, ^9^ More recently, a radiation oncology dedicated Patient Safety Organization (PSO) has been established for the reporting of errors on a national level to reduce the risk of future occurrences.^(10)^

Treatment errors can be broadly classified as failures of software, equipment, or work practices. Concerning work practices, a 10‐year study of voluntary error reporting by Kalapurkal et al.^(4)^ showed that approximately 51% of the errors recorded were related to patient setup and/or delivery. The Pennsylvania Department of Environmental Protection documented and analyzed 37 medical linear accelerator events that were reported from 2004 to 2009, which showed that treatment of an incorrect site was the most frequent event, accounting for nearly half of those reported.^(11)^

Most radiotherapy departments have a number of safeguards to ensure that the correct site is treated. These include indexing the immobilization device to the treatment table, as well as the acquisition and verification of couch parameters within the record‐and‐verify system. Treatment couch parameters — most commonly lateral (lat), longitudinal (lng), and vertical (vrt) positions — document the location of the couch relative to the linac isocenter. By including a tolerance, one can potentially limit the daily variability of the couch, and hence the daily position of the patient, provided he/she is positioned consistently at the same location on the treatment couch.

To our knowledge, there are no published data documenting departmental policies on table position overrides or the magnitude of treatment table tolerances currently being used in radiotherapy. As such, the goal of this study was to survey radiation therapy clinics on the use of table tolerances. The first part of the survey assessed departmental policies detailing override frequency and who has the authority to perform such overrides. The second part of the survey asked respondents to provide data on the table tolerances used in their clinics for specific disease sites. Our hope is that this snapshot of the use of table tolerances will facilitate discussion and enhance patient safety during daily treatment delivery.

## II. MATERIALS AND METHODS

### A. Study population

Full‐member physicists of the American Association of Physicists in Medicine (AAPM) located in North America were sent surveys by email through the SurveyMonkey.com service in December 2014. The AAPM directory (as of 11/2014) was used to identify full‐member physicists. Efforts were made to prevent survey invitations from being sent to multiple physicists at the same institution using the employer name and email address information of AAPM members. To ensure that therapy physicists who wanted to participate in the survey were not excluded unintentionally, in January 2015 information about the survey was posted on the AAPM website general bulletin board with instructions on how to request an invitation to participate.

### B. Survey

A 25‐question treatment overrides and couch tolerances survey was designed to study current patterns of practice among radiation oncology departments in North America (Appendix A). A letter was emailed with a unique link to the survey describing the purpose of the project and emphasizing the confidentiality of the responses. The survey was split into two sections: treatment override practices and couch tolerances. A break was placed between the sections asking participants if they wished to complete the couch tolerances portion of the survey. If the answer was no, participants were taken to the final page of the survey to fill in respondent information.

In the treatment overrides portion of the survey, participants were first asked if couch tolerance overrides are allowed at their institution. If so, participants were subsequently asked details about their institution's override policies, including the typical frequency of overrides, who is allowed to override (e.g., therapists, physicists) and if restrictions differ depending on who performs the override. Participants were also asked if their institution requires an image to be acquired when an override is performed in addition to actions taken when an override is performed (e.g., patient imaged, physics notified). An “other” answer choice was included, allowing respondents the opportunity to identify override actions not included in the choices listed.

The couch tolerances portion of the survey was organized by treatment site: brain/head and neck (H&N), lung, breast, prostate, and abdomen/pelvis. Each site was further divided into IMRT/VMAT and 3D conformal techniques. For each site, participants were asked to select their closest couch tolerance values (vrt, lng, lat) in centimeters from a drop‐down list. Five free‐text couch tolerance group “questions” with the same vrt, lng, lat drop‐down lists followed, allowing respondents to type in any couch tolerance groups that they were unable to specify with the questions provided.

The survey was intentionally designed to be as brief as possible. As a result, in the couch tolerances section of the survey, treatment sites specifically identified were limited to those most common, with similar sites grouped together. However, questions were provided that allowed participants to specify additional sites and/or tolerance groups specific to their institution. Additionally, the survey did not include all treatment parameters that could be overridden —i.e., couch, gantry, and collimator angles — nor did it include the additional parameters found in 6D treatment couches.

### C. Statistics

The Mann‐Whitney U test in Excel (Microsoft Corp., Redmond, WA) was used to evaluate correlations between couch tolerance values and responses from the treatment overrides and respondent information sections of the survey.

## III. RESULTS

A total of 361 individuals responded, of which approximately half participated in the couch tolerances portion of the survey. The survey was sent to a total of 3,153 physicists, giving a response rate of 11% not accounting for invitations sent to non‐therapy physicists, bounced invitations, and invitations not received due to institution spam filters. Participants represented Canada, the District of Columbia (DC), and 40 states. Canada comprised 7% of the responses, and by region of the United States: 24% East, 24% South, 30% Midwest, and 14% West. A majority of the respondents were physicists (97%), which was expected as the distribution list targeted AAPM full‐member physicists in North America. Other participants included physicians, therapists, and nurses. (Results analysis included all responses.) Most of the participants responded on behalf of community or private hospitals (47%), followed by cancer centers (22%), academic centers (20%), private clinics (7%), and government (4%). Of these participants, 50% utilize Aria (Varian Medical Systems, Palo Alto, CA) for their record and verify system, 48% use MOSAIQ (Elekta Inc, Sunnyvale, CA), and 2% use other systems.

### A. Treatment overrides

Eighty‐six percent of respondents’ institutions allow couch tolerance overrides at treatment. Table 1 shows the various combinations of staff members that respondents’ institutions allow to override, with therapist+physicist and therapist‐only being essentially tied for the most common, followed by therapist+dosimetrist+physicist. (Respondents could select all choices that applied.) The “other” (free‐text) responses from the survey all fell into one or more of the provided answer choices (included in Table 1 numbers). Comments included that only more senior therapists (i.e. chiefs, leads, managers) are allowed to override and that, in order for overrides to be performed, multiple people must approve the override, either of the same position or of some combination of a therapist, physician, and physicist. Thirty‐nine percent of respondents impose different restrictions on overrides, depending on the position of the person performing the override. Of the institutions allowing overrides, the most common typical frequency of overrides was daily, with 34% of the responses; followed by weekly, monthly, “other,” and biweekly with 28%, 15%, 15%, and 8% of responses, respectively. The most common “other” response was “rarely”, comprising 5% of the total responses for this question.

Respondents were asked to select what action(s), if any, are taken at their institution when an override is performed. As shown in Table 2, the most common actions that are taken are documentation of the reason for the override (57%), acquiring an image of the patient (53%), and notifying a physicist (31%). Respondents were also allowed to include an “other” free‐text response for this question; common responses included: actions taken vary based on the frequency and/or magnitude of the override, overrides are reviewed by physicists post‐treatment, and patient setup is verified by therapists or chief therapists (without imaging) prior to treatment. Combinations of responses to this question were also analyzed. The top four combinations were: documentation (alone), 13.92%; nothing — treatment continues with overridden parameters (alone), 12.45%; documentation+patient imaged, 11.71%; and patient imaged (alone), 10.62%. A noteworthy result was that 12.45% of respondents answering this question do not take any action and continue treating with the overridden couch parameters.

Participants were also asked if their institutional policy requires an image to be acquired if there is a couch tolerance override. Approximately 43% of sites require an image to be obtained while 40% do not. Seventeen percent of the responses require an image “sometimes,” with the most common responses clarifying that an image is required if the magnitude of the override is greater than a certain amount (e.g., > 1 cm), the case is SBRT/SRS, or the override reason is unclear. Some institutions set their override imaging requirements based on treatment site.

**Table 1:** Percentage of personnel by role who are allowed to perform treatment couch overrides

*Therapist*	*Dosimetrist*	*Physicist*	*Physician*	*Percent*
x		x		24.55%
x				24.19%
x	x	x		19.50%
x	x	x	x	15.16%
		x		6.14%
x		x	x	5.78%
	x	x		2.52%
	x	x	x	1.08%
x	x			0.36%
x			x	0.36%
		x	x	0.36%

**Table 2:** Action(s) taken when an override is performed

*Override Reason Documented*	*Patient Imaged*	*Physics Notified*	*Nothing; Treatment Continues with Overridden Parameters*	*Physician Notified*	*Dosimetry Notified*	*Patient Not Treated That Day*	*Percent of Responses to Question*
x							13.92%
			x				12.45%
x	x						11.71%
	x						10.62%
x	x	x					5.86%
x		x					5.86%
	x		x				5.49%
	x	x					3.66%
x			x				3.3%
x	x	x		x			2.93%
	x			x			2.93%
		x					2.56%
x	x			x			2.2%
		x			x		1.82%
x		x		x			1.82%
x	x	x			x		1.46%
x	x	x		x	x		1.1%
	x	x		x			1.1%
x	x		x				1.1%
x	x				x		0.73%
x		x			x		0.73%
x				x			0.73%
x	x	x	x	x	x	x	0.37%
	x	x	x	x	x		0.37%
	x	x	x		x		0.37%
x	x	x		x		x	0.37%
	x	x	x	x			0.37%
x	x	x	x				0.37%
x	x			x	x		0.37%
	x				x		0.37%
x	x		x	x			0.37%
x		x		x	x		0.37%
x		x	x		x		0.37%
		x		x			0.37%
		x	x				0.37%
x				x	x		0.37%
x			x		x		0.37%
				x			0.37%
*Percent of Responses for Each Action*							
56.8%	54.2%	32.6%	25.6%	16.5%	9.2%	0.7%

### B. Couch tolerances

**Figure 1:**
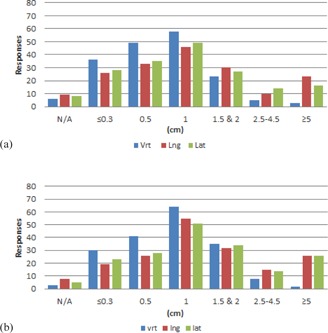
Number of responses vs. individual table tolerance value for (a) IMRT/VMAT and (b) 3D conformal brain/H&N treatments. Vrt=vertical couch tolerance,Lng=longitudinal couch tolerance,Lat=lateral couch tolerance.

**Figure 2:**
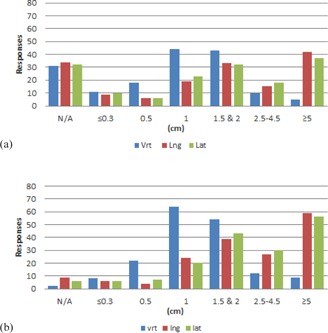
Number of responses vs. individual table tolerance value for (a) IMRT/VMAT and (b) 3D conformal breast treatments. Vrt=vertical couch tolerance,Lng=longitudinal couch tolerance,Lat=lateral couch tolerance.

**Figure 3:**
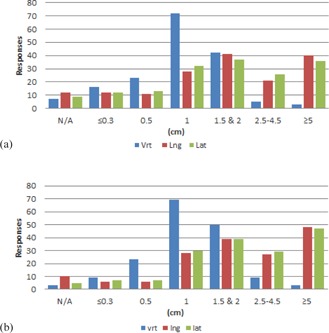
Number of responses vs. individual table tolerance value for (a) IMRT/VMAT and (b) 3D conformal lung treatments. Vrt=vertical couch tolerance,Lng=longitudinal couch tolerance,Lat=lateral couch tolerance.

**Figure 4:**
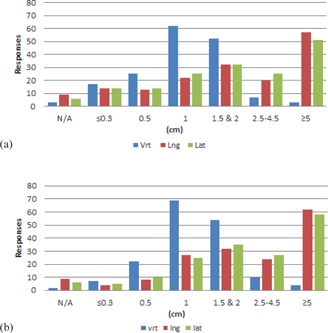
Number of responses vs. individual table tolerance value for (a) IMRT/VMAT and (b) 3D conformal abdomen/ pelvis treatments. Vrt=vertical couch tolerance,Lng=longitudinal couch tolerance,Lat=lateral couch tolerance.

**Figure 5:**
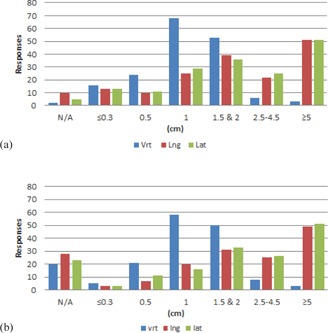
Number of responses vs. individual table tolerance value for (a) IMRT/VMAT and (b) 3D conformal prostate treatments. Vrt=vertical couch tolerance,Lng=longitudinal couch tolerance,Lat=lateral couch tolerance.

**Figure 6:**
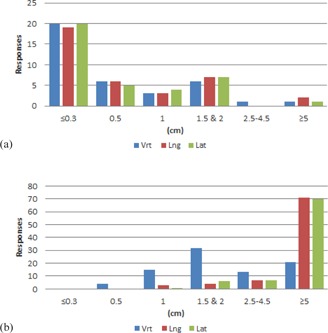
Number of responses vs. individual table tolerance value for (a) SRS/SBRT treatments and (b) 3D conformal clinical setup treatments. Vrt=vertical couch tolerance,Lng=longitudinal couch tolerance,Lat=lateral couch tolerance.

Results from the couch tolerances section of the survey are shown in Figs. 1–6 by number of responses vs. table tolerance. For each site, the values in centimeters in the vrt, lng, and lat drop‐down lists from which participants could select were: N/A (not applicable), 0.0, 0.2, 0.3, 0.5, 1.0, 1.5, 2.0, 2.5, 3.0, 3.5, 4.0, 4.5, and 5.0. Based on the distribution of the results, for ease of review, the couch tolerance values for vrt, lng and lat were binned into the following groups: N/A,≤0.3,0.5,1,1.5&2,2.5–4.5, and ≥5.

A total of 158 responses to the free‐text couch tolerance questions were received. Results relevant to the treatment sites specified in the survey were incorporated into the appropriate site, totaling four for breast and eight for prostate. The remaining responses fell into either “SRS/SBRT” or “Clinical Setup/Mets/Extremities.” A majority of the Clinical Setup/Mets/Extremities responses specified tolerances for electrons, with other groups including unindexed patients, “general,” palliative, and clinical photon setups. Responses with values left blank were omitted from analysis, as were groups with fewer than five results that did not fall into any of the above groups (e.g., craniospinal). (These types of responses amounted to a total of 24.)

For brain/H&N (Fig. 1), the overall distribution of responses for both the IMRT/VMAT and 3D conformal tolerance groups showed a bell shape, with 1 cm being the most common value for vrt, lng, and lat. Higher values of vrt were less common, whereas values of ≥5cm for lng and lat were the most frequent.

Breast (Fig. 2), lung (Fig. 3), abdomen/pelvis (Fig. 4) and prostate (Fig. 5) couch tolerance results (both IMRT/VMAT and 3D conformal) showed the same general trends. The overall distribution of responses for both the IMRT/VMAT and 3D conformal tolerance groups showed a majority of the responses with vrt, lng, and lat values >1cm. The vrt values exhibited a peak at 1 cm for both IMRT/VMAT and 3D conformal, followed by 1.5 & 2 cm. Whereas higher values of vrt were less prevalent, values ≥1cm encompassed the majority of responses for lng and lat. With the exception of the lung IMRT/VMAT group, lng and lat values ≥5cm were the most frequent response within each treatment site group. Additionally, the breast (3D conformal), prostate, and abdomen/pelvis groups exhibited a relatively high frequency of lng and lat values in the ≥5cm bin.

Comparing IMRT/VMAT and 3D conformal groups within each treatment site did not reveal major differences overall. Though, in the IMRT/VMAT group for every site there is a greater amount of vrt, lng, and lat responses in the ≤0.3 and 0.5 cm bins compared to the 3D conformal group of the same site. Of note, for breast, about 20% of the respondents marked IMRT/VMAT as not applicable to their institution, and for prostate about 14% marked 3D conformal as not applicable to their institution.

The free‐text couch tolerance groups, SRS/SBRT and Clinical Setup/Mets/Extremities (Fig. 6), were, unsurprisingly, at opposite ends of the spectrum. The most frequent response for vrt, lng, and lat was ≤0.3cm for the SRS/SBRT group, comprising about half of the responses. The remaining responses were split approximately evenly between 0.5, 1, and 1.5 & 2, with a couple responses in the ≥5cm group. Conversely, for the Clinical Setup/Mets/Extremities group, a majority of responses for lng and lat were ≥5cm. However, the vrt tolerance values were spread out between 0.5 and ≥5, with the majority of responses in the 1.5 & 2 cm bin.

### C. Statistical analysis

There was no statistically significant difference between couch tolerance values based on override frequency and R&V system used. With the exception of the 3D brain lng parameter showing a marginal significance (p=0.055), there was no statistically significant difference between couch tolerance values of academic centers vs. all other institution types.

Statistical significance was found comparing couch tolerance values to if an image is required upon an override, but only for 3D brain lng and lat parameters, with p‐values of 0.026 and 0.022, respectively. The median lng and lat values of those requiring an image upon override were 2 cm; the median lng and lat values of those not requiring an image upon override were 1 cm, illustrating that institutions not requiring an image upon override have a higher proportion of tighter tolerance values than institutions requiring an image.

Statistical significance between the IMRT/VMAT and 3D conformal couch tolerance values was also examined. The only two couch tolerance parameters that exhibited statistically significant differences between IMRT/VMAT and 3D conformal were brain/H&N (lat) and lung (lat), with p‐values of 0.04 and 0.05, respectively. Though the brain/H&N lat and lung lat couch tolerance value distributions have the same median value for IMRT/VMAT and 3D conformal techniques, their overall distributions were skewed. The IMRT/VMAT distributions exhibit a higher percent of tolerance values <1cm than the 3D conformal ones.

## IV. DISCUSSION

In this study, we present the results of the first North American survey conducted on couch overrides and table tolerances. This survey was motivated by an increased frequency of couch parameter overrides that were observed in our clinic. In an attempt to better understand these overrides, and to potentially reduce their frequency, we found little data in the peer‐reviewed literature to provide guidance, and that the selection of table tolerances was often based on anecdotal evidence. As such, we became interested in understanding on a larger scale the individual policies that other clinics have enacted, as well as the magnitude of individual tolerances that were used, with the hope that we and others could benefit from this knowledge.

There are several interesting observations from this survey. The first is the typical frequency of table overrides. Approximately 34% of the clinics reported performing overrides daily. This high frequency of table overrides is concerning, as overrides should provide a “hard stop” in the treatment process with therapists resolving any potential issue prior to initiating treatment. However, when overrides become frequent and routine, this may desensitize individuals such that they do not recognize a treatment anomaly that can have dire consequences for the patient. Establishing appropriate couch tolerance values is therefore a delicate balance: very tight tolerances are likely to catch setup errors, but can result in excessively frequent overrides, while loose tolerances can mask setup errors, but result in less frequent overrides.^(12)^ Establishing tolerance values near the middle of this spectrum is most realistic, but this introduces the potential to miss setup errors. Consistent, well‐understood setup procedures and checks are paramount.

Another observation is that most clinics do not make much of a distinction between IMRT/VMAT and 3D planning table tolerances, as illustrated by only brain/H&N lat and lung lat showing statistically significant differences. The clinical setup tolerance group showed the largest values, with the most frequent being ≥5cm. Such a tolerance may be reasonable for most clinical setups; however, one needs to recognize that patients having clinical setups often have multiple sites being treated simultaneously. If the table is not moved after treating the first isocenter, a large tolerance may permit delivering the second site's beams at the first position. Hence, care must be taken to ensure clinical setups table tolerances are not too large.

Though not as visibly apparent as the clinical setup couch tolerance group, tolerance values of ≥5cm were selected for other sites more frequently than expected. About 10% reported lat and lng table tolerances ≥5cm for brain/H&N and about 25% reported lat and lng table tolerances ≥5cm for the other sites. Several respondents commented that their table tolerance values are large since they rely on imaging, and, interestingly, there were a couple comments that couch tolerances are no longer used due to all patients receiving IGRT. Unfortunately, questions regarding the utilization of IGRT were not included in this survey. Thus, a key topic that needs to be addressed is the role of table tolerances in the IGRT era. If patients are imaged daily, then the exact table position may be less relevant. However, there is the possibility that the image can be interpreted incorrectly and therefore an error in the patient position may go undetected if the couch tolerances are too large.^(12)^ Moreover, not all centers image patients daily. Many institutions have protocols where patients are imaged every other day, or weekly. In these cases, table tolerances are especially important as a safeguard on days when patients are not treated under image guidance.

In our clinic, overrides had become a daily occurrence, prompting an examination of our couch tolerances and policies and the subsequent creation of this survey as a means to confirm safe practices. We enacted a number of steps to reduce the amount of overrides that the therapists perform, including discussing the significance of overrides with the therapists to ensure they are aware of the important safety risks that these alerts attempt to prevent. Additionally, we determined that a number of overrides occurred when immobilization devices were placed at the wrong indexing position on the treatment table. Therefore, after the first treatment, the therapists are to write the index location on the device itself so that it can be more easily positioned identically each treatment. Lastly, we closely examined our table tolerances and found that by increasing our lat and lng parameters a few millimeters (clinically acceptable) we were able to reduce overrides significantly. With these efforts, we observed nearly a 70% reduction in table overrides within the first month. Most significantly, there was a marked reduction in the large longitudinal overrides from the more consistent indexing of the immobilization devices to the treatment table.

Steps were also enacted to ensure the larger overrides (>3cm) being performed are justifiable. Firstly, for any overrides >3cm, the therapists are to document the reason for the override in the patient chart. Knowing documentation is required not only creates a “pause” for the therapists to carefully think through the override, but it also provides a way for physicists to reasonably evaluate the safety of these larger overrides during weekly chart reviews. Additionally, for overrides >3cm, two staff members (therapist, physicist, dosimetrist, and/or physician) must check the patient setup and be in agreement that the override is safe and accurate before treatment occurs. These changes have also helped to reduce the number of overrides as larger overrides are thoroughly examined by multiple people prior to proceeding. However, more importantly, they have helped ensure that patients’ treatments are safer and understood by everyone. Though the changes in our clinic were enacted prior to the survey, the survey results showed that documentation and multiple people approving an override is common practice, reaffirming the validity of our clinic's newly implemented policies. The survey results also confirmed that our couch tolerance values were within the more frequent responses.

The survey had some noteworthy limitations. It was intentionally designed to be short to encourage participation, resulting in less information collected. Knowing the couch tolerances section would be time‐consuming, participants were asked if they wished to participate and were informed they could return to finish at a later time. Providing participants the option to return at a later time or to opt out of the couch tolerances section altogether resulted in fewer responses in the tolerances and respondent information sections of the survey than the overrides section. Another limitation of the couch tolerances section is that 5 cm was the largest couch tolerance available in the drop‐down boxes; however, it became apparent reading through respondent comments that several institutions use larger values. Further stratification could have been achieved with additional drop‐down choices. Additional limitations applicable to all surveys include potential differences in question interpretation among respondents, accidental erroneous responses, and skipped questions.

## V. CONCLUSION

In this study, we have presented the results of a survey on policies related to treatment overrides, as well as provided a summary of table tolerances used in clinics in North America. Based on these results, clinics should review their override policy and table tolerance values in light of the experiences of other institutions presented in this survey to potentially enhance patient safety during daily treatment delivery. Careful attention to these matters is crucial in ensuring the safe and accurate delivery of radiotherapy.

## COPYRIGHT

This work is licensed under a Creative Commons Attribution 3.0 Unported License.

## APPENDIX

### Appendix A: Couch Tolerance Overrides at Treatment

1. Does your institution allow any couch tolerance overrides at the machine at treatment?
  □ Yes
  □ No
2. Who can override? (Please select all that apply.)
  □ Therapist
  □ Dosimetrist
  □ Physicist
  □ Physician
  □ Other (please specify):_____
3. Are there different restrictions on overrides depending on who performs the override?
  □ Yes
  □ No
4. What is the approximate frequency of overrides at your institution?
  □ Daily
  □ Biweekly
  □ Weekly
  □ Monthly
  □ Other (please specify):_____
5. What is done when an override is performed? (Please select all that apply.)
  □ Patient imaged
  □ Physics notified
  □ Dosimetry notified
  □ Physician notified
  □ Override reason documented e.g. reason for override: patient not indexed □ Patient not treated that day
  □ Nothing; treatment continues with overridden parameters
  □ Other (please specify):_____
6. Do you require an image to be acquired if there is an override?
  □ Yes
  □ No
  □ Sometimes (please explain):_____
  Thank you for participating in the first half of this survey. The second half covers couch tolerance values so you will need to have your tolerance tables accessible while filling out this section. We hope the additional time spent will benefit the radiotherapy community as a whole. If you don't have your couch tolerance data accessible at the moment, you can return to this point & complete the survey at a later time.
7. Would you be willing to participate in the couch tolerances portion of the survey? (You can return to this point to finish the survey at a later time.)
  □ Yes
  □ No
***Couch Tolerances***
  Please select your couch tolerance values. If an exact value is not listed, please select the closest one. (Please select N/A for sites or techniques not used at your institution.) If you have additional tolerance groups that are not specified below, please list them & select their couch tolerance values on the next page. You can return to this point & complete the survey at a later time if you don't have your couch tolerance data accessible at the moment.
Brain/H&N (select closest value)
  **Table:** 

*IMRT/VMAT*	*3D Conformal*
Vrt (cm)	
Lng (cm)	{N/A, 0.0, 0.2, 0.3, 0.5, 1.0, 1.5, 2.0, 2.5, 3.0, 3.5, 4.0, 4.5, 5.0}
Lat (cm)
9. Lung (select closest value)
  **Table:** 

*IMRT/VMAT*	*3D Conformal*
Vrt (cm)	
Lng (cm)	{N/A, 0.0, 0.2, 0.3, 0.5, 1.0, 1.5, 2.0, 2.5, 3.0, 3.5, 4.0, 4.5, 5.0}
Lat (cm)
10. Breast (select closest value)
  **Table:** 

*IMRT/VMAT*	*3D Conformal*
Vrt (cm)	
Lng (cm)	{N/A, 0.0, 0.2, 0.3, 0.5, 1.0, 1.5, 2.0, 2.5, 3.0, 3.5, 4.0, 4.5, 5.0}
Lat (cm)
11. Prostate (select closest value)
  **Table:** 

*IMRT/VMAT*	*3D Conformal*
Vrt (cm)	
Lng (cm)	{N/A, 0.0, 0.2, 0.3, 0.5, 1.0, 1.5, 2.0, 2.5, 3.0, 3.5, 4.0, 4.5, 5.0}
Lat (cm)
12. Abdomen/Pelvis (select closest value)
  **Table:** 

*IMRT/VMAT*	*3D Conformal*
Vrt (cm)	
Lng (cm)	{N/A, 0.0, 0.2, 0.3, 0.5, 1.0, 1.5, 2.0, 2.5, 3.0, 3.5, 4.0, 4.5, 5.0}
Lat (cm)
13. Do you have additional couch tolerance groups that you were unable to specify above?
  □ Yes
  □ No
  Please specify your additional tolerance groups & select their couch tolerance values below. If an exact value is not listed, please select the closest one.
14. Other (please specify below)
  Vrt (cm)
  Lng (cm)
  Lat (cm)
  Name of Tolerance Group:_____
15. Other (please specify below)
  Vrt (cm)
  Lng (cm)
  Lat (cm)
  Name of Tolerance Group:_____
16. Other (please specify below)
  Vrt (cm)
  Lng (cm)
  Lat (cm)
  Name of Tolerance Group:_____
17. Other (please specify below)
  Vrt (cm)
  Lng (cm)
  Lat (cm)
  Name of Tolerance Group:_____
18. Other (please specify below)
  Vrt (cm)
  Lng (cm)
  Lat (cm)
  Name of Tolerance Group:_____
***Respondent Information***
  Thank you again for your time completing this survey. Just a few more quick questions.
19. In what state (U.S. only) or country are you located?
  <Select state or country>
20. What type of facility is your institution?
  □ Academic Center
  □ Community or Private Hospital
  □ Private Clinic
  □ Cancer Center
  □ Government
21. What is your role in the department?
  □ Physician
  □ Physicist
  □ Dosimetrist
  □ Therapist
  □ Nurse
  □ Administrator
  □ Other (please specify) :_____
22. What treatment planning system(s) does your institution use?
  □ Eclipse
  □ Pinnacle
  □ BrainLab
  □ Raysearch
  □ Elekta CMS Xio
  □ Elekta CMS Monaco
  □ Other (please specify) :_____Tolerances and Treatment Overrides
23. What record & verify system does your institution use?
  □ Aria
  □ Mosaiq
  □ Other (please specify) :_____
24. Is there anything in your institution's policies, standards, or rules that you were unable to specify in the previous questions? If so, we would greatly appreciate any details you are able to provide.
25. Would you be interested in seeing the results of this survey?
  □ Yes
  □ No
